# The Clinical and Angiographic Profile and Outcomes of Patients With Left Bundle Branch Block (LBBB): An Observational Study From a Tertiary Care Center in North India

**DOI:** 10.7759/cureus.87953

**Published:** 2025-07-14

**Authors:** Suraj Khanal, Jitender Thakur, Basant Kumar, Neelam Dahiya

**Affiliations:** 1 Cardiology, Postgraduate Institute of Medical Education and Research (PGIMER), Chandigarh, IND

**Keywords:** angiographic profile, cag, coronary artery disease, left bundle branch block, pci

## Abstract

Background and objective

Left bundle branch block (LBBB) is a common electrocardiographic abnormality resulting from impaired conduction in both the His-Purkinje system's anterior and posterior left fascicles. LBBB prevalence varies with age, gender, race, and underlying cardiovascular conditions. It affects 0.06-0.1% of the general population, rising to 6-7% in those over 80, and is often detected incidentally in older males. Although once considered a ST‐segment-elevation myocardial infarction (STEMI) equivalent in chest pain, recent data indicate a low risk of myocardial infarction. Diagnosing coronary artery disease (CAD) in these patients is challenging due to limitations of noninvasive tests, often necessitating coronary angiography (CAG) for confirmation. CT angiography is typically the first-line test for patients under 65, while stress nuclear imaging or dobutamine stress echo is preferred in those 65 and older. Certain ECG changes, like ST elevation or depression, may suggest myocardial infarction in LBBB cases. This study aimed to examine various demographic and clinical features, as well as outcomes, in patients presenting with symptomatic LBBB

Methods

This non-randomized, prospective, single-center observational study aimed to evaluate the clinical characteristics, demographic profile, and outcomes of patients presenting with symptomatic LBBB. All enrolled patients underwent CAG, with or without subsequent revascularization, as part of their diagnostic workup.

Results

A total of 200 symptomatic LBBB patients, comprising 104 males (52%), with a mean age of 60.2 ± 11.4 years, were included in the study. Hypertension and diabetes mellitus were the most common comorbidities, observed in 98 (49%) and 68 (34%) patients, respectively. Additionally, 160 (80%) patients exhibited some degree of left ventricular (LV) systolic dysfunction. CAG showed normal findings in 100 patients (50%), obstructive CAD in 78 (39%), and non-obstructive CAD in 22 (11%). The left anterior descending (LAD) artery was the most involved vessel, affected in 70 patients (70%). Among the 78 patients with obstructive CAD, 72 (92.3%) had severe obstruction. The LAD artery was the most affected vessel, observed in 60 patients (76.9%). Percutaneous coronary intervention (PCI) was performed in 42 patients, eight were referred for coronary artery bypass grafting (CABG), and 28 were managed with guideline-directed medical therapy (GDMT). Age ≥50 years (p=0.005), angina (p<0.001), diabetes mellitus (p<0.001), and LV ejection fraction (LVEF) ≤50% (p=0.006) were significantly associated with obstructive CAD. Mortality was also significantly higher in this group (p=0.001). During the six-month follow-up, 12 patients (6%), all over 70 years, died, resulting in an overall survival rate of 94% despite the high-risk profile.

Conclusions

Older patients with LBBB who present with angina, cardiovascular risk factors, and LV systolic dysfunction should be evaluated for CAD by using CAG, especially when troponin markers are elevated (troponin I >0.04 ng/mL).

## Introduction

Left bundle branch block (LBBB) is an intraventricular conduction abnormality usually caused by mechanical or ischemic disturbances affecting the left bundle branch of the heart's conduction system [1]. It results from a disruption in the normal electrical conduction through the left bundle of the His-Purkinje system, leading to a significant alteration in the usual activation sequence of the left ventricle (LV) [2-3]. In LBBB, the right ventricle (RV) is activated before the LV, which adversely affects LV perfusion, increases myocardial workload, and impairs mechanical function [4]. Additionally, LBBB has also been associated with various forms of cardiomyopathy, including dilated, hypertrophic, fibrotic, and infiltrative types, as well as structural heart diseases such as ischemic heart disease and valvular disorders [1].

Epidemiological data show that the incidence and prevalence of LBBB vary across populations, influenced by factors such as underlying cardiovascular conditions, age, gender, and race [1]. Differences in prevalence and underdiagnosis between low- and high-income countries impact clinical outcomes, with social determinants of health playing an increasingly important role in risk stratification [5]. Reported incidence typically ranges from 0.06% to 0.1%, with prevalence rising to 1-5% among individuals over 70 years of age and reaching 6-7% in those over 80 [6]. Moreover, a gender-based study reported an annual incidence of 3.7 per 10,000 in women and 3.2 per 10,000 in men [4]. Eppinger and Rothberger were the first to describe LBBB as a clinical entity on the ECG in canine models over a century ago [7].

In 2009, the American Heart Association Electrocardiography and Arrhythmias Committee, in collaboration with the American College of Cardiology Foundation and the Heart Rhythm Society (AHA/ACCF/HRS), established a widely accepted definition of the LBBB pattern, which was reiterated in 2018 [8-10]. According to this definition, LBBB is characterized by a QRS duration ≥120 milliseconds (ms) in leads I, aVL, V5, and V6, along with a large notched or slurred R-wave [8]. However, in 2011, Strauss et al. proposed a revised criterion, suggesting a higher QRS duration cut-off of ≥140 ms for men and ≥130 ms for women. They also included the presence of a QS or RS pattern in leads V1-V2 and mid-QRS notching or slurring in at least two of the leads V1, V2, V5, V6, I, and aVL [11].

Despite the evaluation of various ECG criteria, there is no established "gold standard" for determining the presence of LBBB, and the interpretation of QRS slurring and notching remains highly subjective [10]. Moreover, ECG interpretation is not fully standardized, and identifying complete LBBB can be challenging [12]. The presence of LBBB often masks or alters other significant electrocardiographic findings, further complicating ECG interpretation. Noninvasive tests, such as CT coronary angiography (CAG), may serve as a first-line approach for evaluating coronary artery disease (CAD), offering an excellent negative predictive value compared to functional strategies and potentially reducing the need for invasive CAG. However, in low-resource settings, limited access to advanced diagnostics hampers the ability to distinguish CAD from non-ischemic causes of LBBB, making CAD detection particularly challenging in these patients [13]. Consequently, CAG is frequently required for definitive diagnosis. Given these diagnostic complexities, the present study aims to assess the clinical characteristics, angiographic findings, and outcomes in patients diagnosed with LBBB.

## Materials and methods

This prospective observational study included patients presenting with symptomatic LBBB to the emergency outpatient and inpatient cardiology departments at the Postgraduate Institute of Medical Education and Research (PGIMER), Chandigarh, India, between December 2022 and June 2024. Eligibility criteria included individuals aged 18 years or older with ECG-confirmed complete LBBB, defined as a QRS duration of ≥120 ms. Participants were required to be hemodynamically stable, exhibit cardiac symptoms, and provide informed consent for study participation. Patients were excluded if they were hemodynamically unstable, unwilling to participate, enrolled in another clinical trial, had incomplete LBBB, had contraindications to CAG, or had a known history of CAD or previous myocardial infarction. The study was conducted in accordance with the ethical principles outlined in the latest edition of the Declaration of Helsinki and applicable Good Clinical Practice (GCP) guidelines. Approval was obtained from the Institutional Ethics Committee under reference number IEC-INT/2022/DM-701.

De-identified data were collected, entered, and analyzed using SPSS Statistics version 24 (IBM Corp., Armonk, NY). Continuous variables were summarized using measures of central tendency (mean, median) and dispersion [standard deviation (SD), interquartile range (IQR)]. Categorical variables were presented as frequencies and percentages. The statistical association between the dependent variable (CAD; present/absent) and various independent variables was assessed using the chi-square test. To evaluate the strength of association between these factors and the outcome (CAD status), unadjusted odds ratios (ORs) with 95% confidence intervals (CIs) were calculated using bivariate binary logistic regression analysis. A similar analysis was conducted with CAD as an independent factor and death as the outcome. A p-value less than 0.05 was considered statistically significant.

## Results

A total of 200 patients with LBBB and cardiac symptoms who met the eligibility criteria were enrolled in the study. The mean age of the study population was 60.21 ± 11.41 years (range: 20-83 years). Notably, 166 patients (83%) were aged 50 years or older. The cohort included 104 males (52%) and 96 females (48%). Hypertension (98 patients; 49%) and diabetes mellitus (68 patients; 34%) were the most frequently observed comorbidities. The baseline characteristics are summarized in Table 1.

**Table 1:** Baseline characteristics of the study population (N=200) SD: standard deviation; ACS: acute coronary syndrome; NSTEMI: non-ST-elevation myocardial infarction; USA: unstable angina; ECG: electrocardiogram; LVEF: left ventricular ejection fraction (calculated using the Simpson’s Biplane method); GLVHK: global left ventricular hypokinesia; RWMA: regional wall motion abnormality; PH: pulmonary hypertension; AS: aortic stenosis; AR: aortic regurgitation; LVEDD: left ventricular end-diastolic diameter; LVESD: left ventricular end-systolic diameter

Variables	Values
Age, years, mean ± SD	60.21 ± 11.41
Sex, n (%)	
Female	96 (48)
Male	104 (52)
Comorbidities, n (%)	
Hypertension	98 (49)
Diabetes mellitus	68 (34)
Hypothyroidism	22 (11)
Dyslipidemia	22 (11)
Others	38 (19)
Presenting complaints, n (%)	
Dyspnoea	146 (73)
Angina	102 (51)
Syncope	16 (8)
Palpitations	10 (5)
ACS, n (%)	
NSTEMI	14 (7)
USA	20 (10)
ECG parameters, mean ± SD	
QRS duration, milliseconds	146.8 ± 17.8
Echocardiographic parameters	
LVEF, % mean ± SD	35 ± 12.5
Normal (≥50%), n (%)	40 (20)
Mild dysfunction (40-49%), n (%)	22 (11)
Moderate dysfunction (30-39%), n (%)	70 (35)
Severe dysfunction (<30%), n (%)	68 (34)
GLVHK, n (%)	104 (52)
RWMA, n (%)	62 (31)
Mitral regurgitation, n (%)	102 (51)
PH, n (%)	26 (13)
AS, n (%)	4 (2)
AR, n (%)	12 (6)
LVEDD, mm, mean ± SD	52.9 ± 7
LVESD, mm, mean ± SD	42.12 ± 9.56

The mean QRS duration among the study population was 146.8 ± 17.8 ms. The average LVEF was 35 ± 12.5%. Severe LV systolic dysfunction, defined as a LVEF below 30%, was observed in 68 patients (34%), while 40 patients (20%) had a normal LVEF. Moderate LV systolic dysfunction was noted in 70 patients (35%), with a total of 160 patients (80%) exhibiting some degree of LV systolic dysfunction. Regional wall motion abnormalities (RWMA) were detected in 62 patients (31%), and global left ventricular hypokinesia (GLVHK) was present in 104 patients (52%). Mitral regurgitation was identified in 102 patients (51%) of the study cohort. The mean left ventricular end-diastolic diameter (LVEDD) was 52.9 ± 7 mm, and the mean left ventricular end-systolic diameter (LVESD) was 42.12 ± 9.56 mm.

Angiographic profile of the study population

The angiographic profile of the study population is summarized in Table 2.

**Table 2:** Angiographic profile of the study population (N=200) CAD: coronary artery disease; LMCA: left main coronary artery; LAD: left anterior descending artery; RCA: right coronary artery; LCx: left circumflex artery

Variables	Values
Access, n (%)	
Radial	176 (88)
Femoral	24 (12)
Dominance, n (%)	
Right	142 (71)
Left	32 (16)
Co-dominant	26 (13)
Pattern of CAD, n (%)	
Obstructive CAD (≥50% stenosis)	78 (39)
Non-obstructive CAD (<50% stenosis)	22 (11)
Normal coronaries	100 (50)
Pattern of Vessel Involvement, n (%)	
LMCA	6 (6)
LAD	70 (70)
RCA	60 (60)
LCx	46 (46)

All patients in the study underwent CAG. Radial access was utilized in 176 cases (88%), and right-dominant coronary circulation was observed in 142 patients (71%). Of the 200 patients evaluated, CAG revealed normal findings in 100 patients (50%), obstructive CAD in 78 (39%), and non-obstructive CAD in 22 (11%). Among those with CAD, the LAD artery was the most involved vessel, affected in 70 patients (70%), followed by the right coronary artery (RCA) in 60 (60%), the left circumflex (LCx) artery in 46 (46%), and the left main coronary artery (LMCA) in 6 patients (6%).

Pattern of coronary vessel involvement in obstructive CAD patients

Table 3 summarizes the pattern of coronary vessel involvement among patients with obstructive CAD.

**Table 3:** Pattern of coronary vessel involvement in obstructive CAD patients (n=78) CTO: chronic total occlusion; LM-TVD: left main triple-vessel disease; TVD: triple-vessel disease; DVD: double-vessel disease; SVD: single-vessel disease; LMCA: left main coronary artery; LAD: left anterior descending artery; RCA: right coronary artery; LCx: left circumflex artery

Variables	Values
Severity of the lesion, n (%)	
Moderate obstruction (50-69%)	6 (7.7)
Severe Obstruction (≥70%)	72 (92.3)
Pattern of the disease, n (%)	
Diffuse	48 (61.5)
Focal	30 (38.5)
Calcification, n (%)	12 (15.4)
CTO, n (%)	4 (5.1)
Number of vessels involved, n (%)	
LM-TVD	6 (7.7)
TVD	22 (28.2)
DVD	18 (23.1)
SVD	32 (41.0)
Type of vessel involved, n (%)	
LMCA	6 (7.7)
LAD	60 (76.9)
RCA	50 (64.1)
LCx	42 (53.8)

Among the 78 patients diagnosed with obstructive CAD, six (7.7%) had moderate obstruction, whereas the majority (72, 92.3%) had severe obstruction. More than half of the patients with obstructive CAD showed diffuse coronary involvement. Extensive calcification was noted in 15.4% of cases, and chronic total occlusion (CTO) was observed in 5.1% of patients. Regarding the number of vessel involvement, single-vessel disease (SVD) was seen in 41% of patients, triple-vessel disease (TVD) in 28.2%, double-vessel disease (DVD) in 23.1%, and left main (LM) with TVD in 7.7%. The LAD artery was the most frequently affected vessel, involved in 76.9% of patients, followed by the RCA in 64.1%, the LCx artery in 53.8%, and the LMCA in 7.7% of patients. Among the 78 patients diagnosed with obstructive CAD, 42 underwent PCI with drug-eluting stents (DES), eight were referred for CABG, and the remaining 28 received GDMT. Of the 68 patients with severe LV systolic dysfunction (LVEF <30%), 30 received an automatic implantable cardioverter-defibrillator (AICD), four were implanted with a cardiac resynchronization therapy (CRT) device that included a defibrillator (CRT-D), and the remaining 34 were managed with GDMT.

Association between clinical variables and obstructive CAD

Table 4 summarizes the association between clinical variables and obstructive CAD.

**Table 4:** Association of clinical variables with obstructive CAD ^*^Statistically significant; ^¥^chi-square test; ^¶^bivariate binary logistic regression LVEF: left ventricular ejection fraction; CAD: coronary artery disease; CI: confidence interval

Variables	CAD (n=78)	No CAD (n=122)	P-value^¥^	Odd’s ratio (95% CI)^¶^
Age, years				
<50	6 (7.5%)	28 (22.9%)	0.005^*^	1.0 (Reference)
≥50	72 (92.3%)	94 (77.1)		3.6 (1.3, 11.1)
Angina				
Absent	24 (30.8%)	98 (80.3%)	<0.001^*^	1.0 (Reference)
Present	54 (69.2%)	24 (19.7%)		3.6 (1.3, 11.1)
Diabetes mellitus				
No	40 (51.3%)	92 (75.4%)	<0.001^*^	1.0 (Reference)
Yes	38 (48.7%)	30 (24.6%)		3.6 (1.3, 11.1)
LVEF				
>50%	8 (10.3%)	32 (26.2%)	0.006^*^	1.0 (Reference)
≤50%	70 (89.7%)	90 (73.8%)		3.6 (1.3, 11.1)

Individuals aged ≥50 years exhibited a significantly higher incidence of obstructive CAD (p=0.005). A strong statistical association was also observed between the presence of angina (p<0.001) and diabetes mellitus (p<0.001) and the development of obstructive CAD. Additionally, an LVEF of ≤50% was linked to an increased likelihood of CAD (p=0.006). The odds of having CAD were 3.6 times higher among patients aged ≥50 years, those with angina, diabetes mellitus, and a LVEF ≤50%, compared to those without these factors. Mortality was also significantly higher in the obstructive CAD group (p=0.001). The odds of death were 8.8 times higher in patients with CAD compared to those without CAD (Table 5).

**Table 5:** Association between obstructive CAD and death as the outcome ^*^Statistically Significant; ^¥^chi-square test; ^¶^bivariate binary logistic regression CI: confidence interval; CAD: coronary artery disease

Variable	Death		P-value	Odds ratio (95% CI)
	Yes (n=12)	No (n=188)		
CAD				
No	2 (16.7%)	120 (63.8%)	0.001^*^	1.0 (Reference)
Yes	10 (83.3%)	68 (36.2%)		8.8 (1.8, 84.2)

Follow-up and outcomes

All 200 patients were followed up in the outpatient department (OPD) for up to six months. During this period, 12 patients (6%) died. All deceased patients were above 70 years of age, comprising eight males and four females. The causes of death included acute coronary syndrome (ACS) and acute decompensated heart failure (ADHF) in four patients each, and sudden cardiac death (SCD) and refractory ventricular tachycardia (VT) in two patients each. Consequently, the overall six-month survival rate was 94% (188 patients), despite being a high-risk population under specialized medical care. Among those who died, 50% had both diabetes mellitus and hypertension, and all had an LVEF of less than 40%. Of the 12 patients, 10 had obstructive CAD and two had non-obstructive CAD. In all patients with obstructive CAD, the LAD artery was involved: four had isolated LAD lesions, two had LM-TVD, 2 had DVD, and 2 had TVD. Among these 10 patients, only two underwent PCI, two were referred for CABG, and the remaining six were managed with GDMT.

## Discussion

As the term implies, LBBB is characterized by a distinct QRS pattern on an ECG, reflecting a conduction delay or block within the left His-Purkinje pathway [14]. Initially regarded as a purely electrocardiographic conduction abnormality, LBBB has evolved into a recognized clinical entity that poses various diagnostic and therapeutic challenges for clinicians [2,15-16]. Renewed interest in LBBB over the past decade has largely been driven by the advent of CRT, with mounting evidence demonstrating a significantly higher response rate among patients exhibiting LBBB-type QRS morphology [12]. Common causes of LBBB include cardiomyopathy (ischemic and idiopathic), acute myocardial infarction, primary conduction system disease, long-standing hypertension, valvular heart disease, particularly aortic valve disease, and myocarditis. Conventional coronary angiography is often required to confirm the diagnosis, as noninvasive tests have limited utility in patients with LBBB [17]. While LBBB was once viewed as a benign, incidental finding with limited clinical relevance, emerging research increasingly implicates it in structural and functional cardiac impairments. Nonetheless, some clinicians may still consider it incidental in specific clinical contexts [18].

A meta-analysis assessing the diagnostic accuracy of three key noninvasive modalities (exercise ECG, stress echocardiography, and myocardial perfusion imaging) for detecting CAD in patients with LBBB found that exercise ECG and myocardial perfusion imaging had the highest sensitivity (83.4% and 82.1%, respectively). In contrast, stress echocardiography demonstrated the highest specificity (88.7%) [12]. Overall diagnostic accuracy was highest for stress echocardiography (84.4%) and lowest for exercise ECG (66.4%) [12]. LBBB significantly influences both clinical management and the interpretation of diagnostic investigations [12]. Nonetheless, the prognostic significance of LBBB in asymptomatic individuals remains a subject of ongoing debate. LBBB, even without clinically evident heart disease, is linked to a higher risk of new-onset heart failure and cardiovascular mortality, and should be considered a “cardiac clinical entity” rather than merely an ECG finding [19]. Further research is needed to assess whether additional diagnostic evaluation or early intervention in asymptomatic LBBB patients can reduce cardiovascular morbidity and mortality [20]. This study aims to evaluate the clinical and angiographic characteristics, as well as the outcomes, of patients with LBBB.

In our study, the baseline characteristics of patients with LBBB were consistent with findings from both Indian and international research [19,21-24]. The mean age of participants was 60.21 ± 11.41 years (range: 20-83 years), with a slight male predominance (52% males vs. 48% females). These demographics align with earlier studies, including one that reported a mean age of 57.2 ± 11.1 years [21] and an Indian observational study that documented a mean age of 62.25 ± 13.75 years, with males accounting for 53.45% of the population [19]. Another study indicated the mean age of LBBB onset to be around 65 years [25], while prospective population-based research reported the average age at diagnosis as 70 ± 10 years for men and 68 ± 11 years for women, highlighting a strong correlation between LBBB prevalence and advancing age [26]. Additionally, other cohorts have shown LBBB to be approximately twice as prevalent in men compared to women [1]. Notably, in our study, 83% of participants were aged 50 years or older. This is in line with previous data indicating that LBBB prevalence increases from less than 1% at age 50 to approximately 6% by age 80 [12]. In this study population, hypertension (49%) and diabetes mellitus (34%) emerged as the most prevalent comorbidities among patients with LBBB. These findings are comparable to those of an Indian observational study, which reported that 50.86% of patients with LBBB had hypertension and 30.17% had diabetes mellitus [19]. Similarly, another study identified hypertension as the most common chronic condition (73.5%), followed by CAD (47.1%) and dyslipidemia (39.7%) in patients with LBBB [27].

The mean QRS duration in our study population was 146.8 ± 17.8 ms. This is consistent with findings from a strain-based staging classification study on LBBB-induced cardiac remodeling, which reported a mean QRS duration of 152 ± 16.2 ms across different stages of LBBB (Stage LBBB-1: 149 ± 14.1 ms; Stage LBBB-2: 150 ± 15.8 ms; Stage LBBB-3: 161 ± 13.5 ms; Stage LBBB-4: 164 ± 17.6 ms) [22]. The study also demonstrated a significant increase in QRS duration with advancing LBBB stages (p<0.001) [22]. Additionally, a case report described a 72-year-old female patient with a baseline ECG showing classic LBBB and a QRS duration of 180 ms [13]. In this study, the average LVEF was 35 ± 12.5%, consistent with previous findings that reported a mean baseline LVEF of 30.5 ± 6.7% before left bundle branch pacing (LBBP) [23]. A staging study demonstrated a progressive decline in LVEF with advancing stages, reporting a mean LVEF of 47 ± 13.6% overall, ranging from 56 ± 9.5% in Stage LBBB-1 to 25 ± 7.5% in Stage LBBB-4. Notably, 23% of patients had an LVEF ≤35%, and in Stage LBBB-4, this proportion increased to 96% [22]. Similarly, a case study described a 72-year-old woman with typical LBBB and an LVEF of 32% [13]. In the present study, approximately one-third of patients had severe systolic dysfunction (LVEF <30%), another third had moderate dysfunction, and 20% had preserved LVEF.

These findings are comparable to those of an Indian observational study, where 62 patients (53.44%) exhibited LV systolic dysfunction, categorized as mild in 14.51%, moderate in 16.12%, and severe in 69.35% of those affected. Additionally, 17 patients had a normal cardiovascular profile [19]. Moreover, previous research has shown that patients with advanced LBBB stages tend to have significantly larger LVESD and LVEDD (p<0.001) [22]. In our study, RWMA were observed in 31% of patients. However, it is important to note that detecting RWMA in the presence of LBBB can be diagnostically challenging due to altered ventricular activation patterns [12]. In our study, the mean LVEDD was 52.9 ± 7 mm, and the mean LVESD was 42.12 ± 9.56 mm. Similar values have been reported in earlier studies, with one indicating an LVEDD of 55.7 ± 2.7 mm [23], and another showing an LVEDD of 64 ± 10 mm and an LVESD of 53 ± 10 mm [28]. Additionally, in a study assessing echocardiographic findings in LBBB patients, those with left ventricular hypertrophy (LVH) had significantly larger LVEDD and LVESD values compared to those without LVH (57.5 vs. 48.0 mm, p=0.001; and 43.5 vs. 34.0 mm, p=0.004, respectively) [27].

The angiographic profile in our study revealed that 50% of patients had normal epicardial coronary arteries, 39% had obstructive CAD, and 11% had non-obstructive CAD. These findings are supported by earlier studies reporting that approximately 48-56% of patients develop overt CAD either concurrently with or following the onset of LBBB [29-30]. Another study similarly observed that 64% of patients had normal angiograms, while 36% showed evidence of vessel disease [31]. Among patients with CAD in our cohort, the LAD artery was the most affected vessel (76.9%), followed by the RCA (64.1%), LCx (53.8%), and the LMCA (7.7%). This pattern is consistent with a prior study in which, among 27 patients, the most frequently involved isolated vessels were the LAD (26.0%), RCA (11.1%), and LCx (3.7%) [27,30]. While several epidemiological studies have implicated CAD as a leading cause of LBBB, others have found no significant association between the two [21]. Furthermore, in our study, among the 78 patients diagnosed with obstructive CAD, 92.3% (n=72) had severe obstruction, whereas 7.7% (n=6) had mild obstruction. In terms of vessel involvement, SVD was observed in 41% of patients, TVD in 28.2%, DVD in 23.1%, and LM with TVD in 7.7%. These findings are consistent with a previous study that reported SVD in 37 patients (31.89%), DVD in 23 patients (19.82%), TVD in nine patients (7.75%), and normal coronary arteries in seven patients (6.03%) following CAG [19].

We also evaluated the association between clinical variables and obstructive CAD. The analysis revealed that individuals aged 50 years or older had a significantly higher incidence of obstructive CAD (p=0.005). Moreover, a strong statistical association was observed between the presence of angina (p<0.001) and diabetes mellitus (p<0.001) and the occurrence of obstructive CAD. These findings are consistent with a previous study in which multivariate regression analysis identified age, gender, diabetes mellitus, dyslipidemia, and typical angina as significant predictors of obstructive CAD [21]. Additionally, Boyle and Fenton reported that 88% of patients with LBBB were aged 50 years or older, and 69% of these individuals had CAD and/or hypertension [19,32]. The results of this study indicated that a LVEF of ≤50% was significantly associated with an increased likelihood of CAD (p=0.006), and mortality was notably higher among patients with obstructive CAD (p=0.001). Supporting this, a previous study reported that LBBB is associated with a 2.9-fold increased risk of cardiovascular mortality and a 1.4-fold increased risk of all-cause mortality [1]. Additionally, patients with LBBB undergoing nuclear testing for suspected CAD were found to have a 50% higher risk of all-cause mortality [1].

In our study, during a six-month follow-up period, 12 patients (6%) died. All deceased individuals were over 70 years of age, including eight males and four females. These findings are supported by a previous study on patients with LBBB and septal flash, which reported that over a 12-month follow-up, nine patients (4%) died, 50 (21%) underwent CRT, one (0.5%) received a pacemaker, and one (0.5%) exhibited modest QRS narrowing [22]. Another study found that patients in the LBBB group experienced greater LVEF reduction (37% vs. 30%) and a higher mortality rate over a five-year follow-up, with a hazard ratio (HR) of 1.17 (95% CI: 1.00 to 1.36) [33]. The Framingham study also highlighted the prognostic significance of LBBB, indicating a substantially elevated risk of cardiovascular death, with 50% of affected individuals dying within 10 years of diagnosis [29]. More evidence has further established LBBB as an independent predictor of adverse cardiovascular outcomes, including a 2.9-fold increased risk of myocardial infarction [26], a 3.08-fold increased risk of heart failure-related mortality, and a 10-fold increase in the incidence of sudden cardiac death, particularly among individuals aged 50 years and older [34].

Limitations

The main limitations of this study include the absence of a control group, a relatively small sample size, and a short follow-up duration of only six months, which may affect the generalizability and robustness of the findings. Additionally, the study's external validity is limited, as the results may not apply to populations with different ethnicities, socioeconomic backgrounds, or healthcare systems. The focus on only symptomatic LBBB patients further restricts the applicability of the findings to those with incidental or asymptomatic LBBB. Moreover, the six-month follow-up period may be insufficient to capture important long-term outcomes such as CRT implantation, heart failure hospitalizations, or delayed cardiovascular mortality. Future studies involving larger cohorts with diverse populations and extended follow-up are warranted to better evaluate long-term outcomes in this patient population.

## Conclusions

LBBB indicates underlying heart disease and is associated with reduced left ventricular function and poorer prognosis. It may indicate ischemic heart disease, cardiomyopathy, or other cardiac conditions, and is associated with a higher risk of heart failure and mortality. This study uniquely focuses on patients with LBBB presenting to a single tertiary care center in North India for one and a half years, providing valuable regional data on a condition often underrepresented in the literature. Unlike many prior studies, which either include asymptomatic individuals or do not stratify based on clinical presentation, this study specifically examines symptomatic patients and follows them for six months post-evaluation. The findings suggest that patients with LBBB aged over 50 years, especially those presenting with angina, diabetes mellitus, and reduced LVEF (≤50%), have a significantly higher likelihood of obstructive CAD and warrant CAG for appropriate evaluation. By identifying high-risk subgroups, the study underscores the potential benefits of early diagnosis and timely intervention in reducing cardiovascular morbidity and mortality. Despite the limited follow-up duration, the study highlights the critical need for comprehensive medical management in this population and adds to the scarce data from low-resource settings, making its insights both clinically relevant and regionally significant. Furthermore, CAD testing in LBBB patients should be based on a comprehensive risk assessment, including symptoms, demographics, and cardiovascular risk factors, rather than the presence of LBBB alone. In the absence of definitive ischemic evidence (e.g., elevated troponin or ischemic symptoms), noninvasive evaluations like coronary CT angiography should be considered.
